# Expression of concern: Development of a chromium oxide loaded mesoporous silica as an efficient catalyst for carbon dioxide-free production of ethylene oxide

**DOI:** 10.1039/d6ra90149b

**Published:** 2026-10-14

**Authors:** Muhammad Maqbool, Toheed Akhter, Sadaf Ul Hassan, Asif Mahmood, Waheed Al-Masry, Shumaila Razzaque

**Affiliations:** a Department of Chemistry, University of Management and Technology, C-II Johar Town 54770 Lahore Pakistan toheed.akhter@umt.edu.pk; b Department of Chemistry, COMSATS University Islamabad Lahore Campus Lahore Pakistan; c Department of Chemical Engineering, College of Engineering, King Saud University Riyadh 11421 Saudi Arabia; d Institute of Physical Chemistry, Polish Academy of Sciences Kasprzaka, 44/51 01-224 Warszawa Poland srazzaque@ichf.edu.pl

## Abstract

Expression of concern for ‘Development of a chromium oxide loaded mesoporous silica as an efficient catalyst for carbon dioxide-free production of ethylene oxide’ by Muhammad Maqbool *et al.*, *RSC Adv.*, 2023, **13**, 32424–32432, https://doi.org/10.1039/d3ra05858a.

*RSC Advances* is publishing this expression of concern in order to alert readers that concerns have been raised regarding the reliability of the GC chromatogram presented in Fig. 5a. An expression of concern will continue to be associated with the article until a conclusive outcome is reached.

Laura Fisher

7^th^ October 2026

Executive Editor, *RSC Advances*

